# Examining the Role of Task Requirements in the Magnitude of the Vigilance Decrement

**DOI:** 10.3389/fpsyg.2018.01504

**Published:** 2018-08-20

**Authors:** Daniel Gartenberg, Glenn Gunzelmann, Shiva Hassanzadeh-Behbaha, J. Gregory Trafton

**Affiliations:** ^1^Department of Biobehavioral Health, The Pennsylvania State University and Proactive Life LLC, New York, NY, United States; ^2^Airman Systems Directorate, United States Air Force Research Laboratory, Dayton, OH, United States; ^3^Interdisciplinary Program in Neuroscience, Georgetown University, Washington, DC, United States; ^4^United States Naval Research Laboratory, Washington, DC, United States

**Keywords:** vigilance, eye tracking, simultaneous task, successive task, vigilance decrement, resource control theory, microlapse

## Abstract

The vigilance decrement in sustained attention tasks is a prevalent example of cognitive fatigue in the literature. A critical challenge for current theories is to account for differences in the magnitude of the vigilance decrement across tasks that involve memory (successive tasks) and those that do not (simultaneous tasks). The empirical results described in this paper examine this issue by comparing performance, including eye movement data, between successive and simultaneous tasks that require multiple fixations to encode the stimulus for each trial. The findings show that differences in the magnitude of the vigilance decrement between successive and simultaneous tasks were observed only when a response deadline was imposed in the analysis of reaction times. This suggests that memory requirements did not exacerbate the deleterious impacts of time on task on the ability to accurately identify the critical stimuli. At the same time, eye tracking data collected during the study provided evidence for disruptions in cognitive processing that manifested as increased delays between fixations on stimulus elements and between encoding the second stimulus element and responding. These delays were particularly pronounced in later stages of encoding and responding. The similarity of the findings for both tasks suggests that the vigilance decrement may arise from common mechanisms in both cases. Differences in the magnitude of the decrement arise as a function of how degraded cognitive processing interacts with differences in the information processing requirements and other task characteristics. The findings are consistent with recent accounts of the vigilance decrement, which integrate features of prior theoretical perspectives.

## Introduction

Power plant workers, baggage handlers, air traffic controllers, military personnel, and pilots all have jobs that require maintaining attention for prolonged periods of time and detecting relatively rare, but critical, events or stimuli. As a result, they all rely fundamentally on sustained attention, or vigilance. Sustained attention tasks are becoming increasingly common as technology takes over many of the moment-to-moment activities required to perform an assortment of tasks in modern society, relegating human operators to roles emphasizing supervisory control.

Sustained attention tasks put pressure on the human cognitive system. In general, this is not because the tasks themselves are complex or difficult to perform. Instead, it is the maintenance of attention on a relatively mundane or monotonous task that creates the difficulty, leading to performance degradation over time. In the scientific literature, this phenomenon is referred to as the vigilance decrement, and is investigated using long-duration tasks (40 min to multiple hours) that require the detection of rare critical events in a continuous stream of otherwise repetitive stimuli. The vigilance decrement is defined as the decreased probability of detecting critical trials in such tasks as time on task increases (e.g., Mackworth, 1948; Davies and Parasuraman, 1982; Warm et al., 2008). A substantial empirical literature has demonstrated that the magnitude of the vigilance decrement is impacted by a variety of task factors, including the stimulus presentation time, the degree of memory involved in the task, stimulus presentation rate, and sensory modality (see Davies and Parasuraman, 1982 for a review).

The dominant theory for explaining the effects of fatigue and time-on-task on sustained attention performance is Resource Theory. According to Resource Theory, cognitive activity depletes information processing resources, which must be replenished. It is theorized that sustained attention tasks place particularly high demands on some resources, most notably attention, leading to breakdowns in cognitive performance that produce the vigilance decrement (Davies and Parasuraman, 1982; Warm et al., 2008). In support of this account, research has shown that, even though vigilance tasks are frequently quite simple, they have high workload due to the need to continuously focus attention on repetitive, generally uninteresting stimuli (Warm et al., 1996, 2008).

The other major theory in the sustained attention literature is Mindlessness Theory (Robertson et al., 1997; Manly et al., 1999). This theory posits that the vigilance decrement is not caused by the depletion of resources due to stress, but instead, is a consequence of the under-arousing nature of sustained attention tasks. According to this theory, repetitive tasks like sustained attention tasks promote automaticity in responding to the more common non-target stimuli. With increased routinization, greater attentional control is required to suppress the automated response and provide the correct response for rare stimuli. This becomes more difficult with time on task, resulting in the vigilance decrement. Mindlessness Theory is supported by findings showing that various factors like increased task monotony, traumatic brain injury, and greater absent mindedness in participants lead to larger vigilance decrements on sustained attention tasks (Robertson et al., 1997; Manly et al., 1999).

Related in some ways to mindlessness theory are two other accounts for the vigilance decrement. The first implicates boredom as a cause for the degradations in performance (e.g., Malkovsky et al., 2012; Danckert and Merrifield, 2018). The theoretical position is that the mundane nature of sustained attention tasks challenges executive functioning mechanisms to engage sufficiently to perform the task, despite the possibility that individuals are motivated to do well. Another account emphasizes the “opportunity costs” of engagement with mundane tasks (Kurzban et al., 2013). In this case, emphasis is placed on competition for resources associated with executive function at any given point in time. Unlike Resource Theory, this account does not propose that these resources are depleted over time, but rather that the allocation of resources among competing options shifts away from the sustained attention task as time on task increases.

Recently, Thomson et al. (2015) have proposed an account that seeks to reconcile Resource Theory and Mindlessness Theory, which they refer to as Resource-Control Theory. Their proposal is that executive control operates to maintain attention on the task at hand, but that doing so requires effort. Over time, maintaining attention depletes executive control resources, which leads to the experience of mind-wandering. In other words, as executive control breaks down, cognitive resources are shifted to off-task activities. In essence, this account proposes that depletion of executive control resources is the underlying cause of the vigilance decrement, while mind-wandering is the major consequence.

We have proposed a computational model to account for the vigilance decrement (Veksler and Gunzelmann, 2018), which is generally consistent with Resource-Control Theory. The model accounts for the vigilance decrement by introducing breakdowns in goal-directed processing that become more frequent as time on task increases. We refer to these disruptions as microlapses, which are implemented in the model as disruptions lasting on the order of 10’s of milliseconds. A small number of microlapses leads to increases in response times. However, as the likelihood of a microlapse increases, much more substantial disruptions in performance are possible, especially in time-critical tasks. In the next section, we discuss hypotheses that can be derived from this model and the Resource-Control Theory, which serve as the motivation and foundation for the experiment presented below.

### Identifying the Underlying Mechanism Responsible for the Vigilance Decrement

In most resource theories, depleted resources impact sensitivity to the target (see Thomson et al., 2015). In contrast, our model and Resource-Control Theory hypothesize that the critical resource relates to executive control and the capacity to maintain focused attention. Thus, the depleted resource does not affect sensitivity to the stimulus, but rather impacts attentional focus in central cognition. As a result, these accounts are consistent both with subjective reports of workload and effort in vigilance task performance (e.g., Hitchcock et al., 2003), and with the subjective experience of distraction or off-task mind wandering (e.g., Manly et al., 1999).

Resource-Control Theory and the model in Veksler and Gunzelmann (2018) also posit a single resource associated with executive control. This is a crucial distinction, since one of the primary empirical findings supporting Resource Theory relates to a distinction between vigilance tasks that require participants to store critical trial information in memory (successive tasks), and tasks where all of the information needed for a judgment is available on the screen for any given trial (simultaneous tasks). This distinction is generally used to argue for the existence of multiple resources that can be depleted in vigilance tasks (See et al., 1995; Caggiano and Parasuraman, 2004).

In a typical simultaneous task, participants are presented with a stimulus consisting of multiple features or elements, for instance a pair of line segments. Target stimuli are defined by some relationship between stimulus elements, for instance two points that are the same distance from an object versus two points that have different distances (Szalma et al., 1999). In contrast, successive tasks are defined by the requirement to hold a representation of a comparison stimulus in memory. To identify a target stimulus in a successive task, one must compare the stimulus to the one stored in memory. For instance, a single dot that is either closer or farther from an object, where the representation for what distinguishes close and far must be stored in memory (Szalma et al., 1999).

The original proposal that there is a difference between simultaneous and successive judgments in vigilance came from a meta-analysis of studies, which showed strong intra-task type correlations of performance decrements, coupled with low inter-task type correlations (Davies and Parasuraman, 1982). This distinction between simultaneous and successive vigilance tasks aligns with a common distinction in Resource Theory, mentioned above, between resources associated with the supervisory attentional network and resources associated with memory (Davies and Parasuraman, 1982; See et al., 1995). Somewhat circularly, however, much of the evidence for this distinction comes from experiments that have manipulated the involvement of memory using tasks that contrast successive-type judgments with simultaneous-type judgments (Davies and Parasuraman, 1982; See et al., 1995; Warm and Dember, 1998; Caggiano and Parasuraman, 2004; Helton and Russell, 2011, 2013). For example, tasks that require a successive judgment typically show a steeper vigilance decrement (Davies and Parasuraman, 1982; See et al., 1995; Warm and Dember, 1998; Caggiano and Parasuraman, 2004).

A potential concern with existing results relates to standard methodologies that are applied in the vigilance literature. Specifically, to equate task conditions for a vigilance study, stimulus presentation duration is typically held constant to avoid known influences of this factor on the magnitude of the vigilance decrement (e.g., Baker, 1963). However, successive tasks that involve memory requirements may produce concomitantly longer response times than for simultaneous tasks due to the differences in the information processing requirements. That is, accessing information from memory and making a “cognitive” comparison may take longer than making a “perceptual” judgment. If this is the case, then successive tasks may show steeper declines in accuracy with time on task as a consequence of the timing of stimulus presentations and other task factors (c.f., Gartenberg et al., 2014). If this is the case, it may be possible to account for the difference between simultaneous and successive vigilance task performance without having to hypothesize that multiple resources are involved.

There is empirical evidence to support the hypothesis that successive tasks result in a steeper vigilance decrement because they typically take longer to perform. In a vigilance task where the simultaneous task involved an uncharacteristically high perceptual demand, the simultaneous task induced a steeper vigilance decrement than the successive tasks (Grubb et al., 1995). Grubb et al. (1995) observed that most simultaneous/successive judgment tasks involve simple stimuli. By manipulating the complexity of symbols that were presented as an aircraft display, and whether or not these symbols required a successive or simultaneous judgment, Grubb et al. (1995) found that the vigilance decrement was more severe for successive tasks than simultaneous tasks in some cases.

Compounding the influence of stimulus durations is the standard use of response cutoffs in vigilance tasks (e.g., Hitchcock et al., 1999, 2003; Szalma et al., 2006). Previous studies have employed cutoffs to ensure that only responses associated with a critical signal were scored as correct. Yet if successive tasks typically take longer than simultaneous tasks then a response cutoff may differentially impact successive tasks in typical vigilance paradigms. While it may not always be possible to detect how long a memory process takes, a response cutoff could have contributed to performance differences reported between simultaneous and successive tasks by treating some slower responses as errors (Gartenberg et al., 2015).

The focus of the experiment described next is to explore methodological issues that may impact performance on vigilance tasks. We do this by equating successive and simultaneous task stimuli for perceptual difficulty and adjusting presentation times based on how long it takes for the stimuli to be processed. In addition, we analyze the data with and without a response cutoff. If no difference in the vigilance decrement is found between successive and simultaneous task conditions when these modifications are made, it would support the conclusion that the same mechanisms are responsible for the vigilance decrement in both tasks. This would provide support for Resource-Control Theory and our model.

The model in Veksler and Gunzelmann (2018) allows for more detailed predictions about changes in performance associated with the vigilance decrement. One of the important mechanisms in the model produces compounding effects of the vigilance decrement within individual trials. Specifically, when a microlapse occurs, it becomes more likely that additional microlapses will occur later in that trial. In the tasks used here, each trial includes two stimulus elements that must be encoded. Responses must be made for critical trials based on features of those stimulus elements. The eye tracking data allows us to segment trials into encoding times for each item, followed by the time needed to make a response for critical trials. The model predicts that the vigilance decrement should lead to larger performance decrements for later stages of the trial (e.g., the time between encoding the second stimulus element and responding) relative to earlier stages (e.g., the time between stimulus presentation and encoding the first stimulus element). Moreover, if the same mechanisms are influencing performance changes in simultaneous and successive task variants, there should be a similar rate of cognitive slowing in both tasks at each stage of processing the stimuli.

## Experiment

### Method

#### Participants

Sixty George Mason University undergraduate students participated for course credit: 30 participants were assigned to the successive task condition and 30 participants were assigned to the simultaneous task condition. Participation was voluntary and all participants provided informed consent prior to participation. During the study, participants’ cell phones were taken away to remove that potential source of distraction. Data from 46 females and 14 males were analyzed. The average age of participants was 21.32 years old with a standard deviation of 4.95 years. All participants had normal or corrected-to-normal vision. One participant was eliminated because the experimenter forgot to take their cell phone and two participants were eliminated due to errors in the experiment software – resulting in a total of 57 participants in the results presented below, 28 in the successive task condition and 29 in the simultaneous task condition.

Eye data for three participants were eliminated due to an error in the software. Fifty-four participants’ eye data were analyzed, 26 for the successive task condition and 28 for the simultaneous task condition. If there were no fixations recorded for a given trial, that trial was not included in the eye data analysis. This resulted in the elimination of 11.11% of the trials.

#### Materials

The stimuli for the study consisted of pairs of letters. Each trial contained 2 letters, consisting of either 2 p’s, 2 d’s, or one of each. Letters appeared in either red or blue on a white background. The letters were presented at canonical positions on a clock face with a diameter of 31.8 cm. The clock face was not presented to participants. In addition to the 2 letters, a filled red circle with a diameter of 3.8 cm was presented at the center of the screen on each trial. Each letter was 0.32 cm high and 0.24 cm wide. Locations for the letters for each trial were chosen randomly, with the constraint that they must be separated by at least three positions around the clock face. As a result, letters were separated by 17.26–25.87° of visual angle. This spacing ensured that eye movements were necessary to encode the identity of the letters.

**FIGURE 1:**
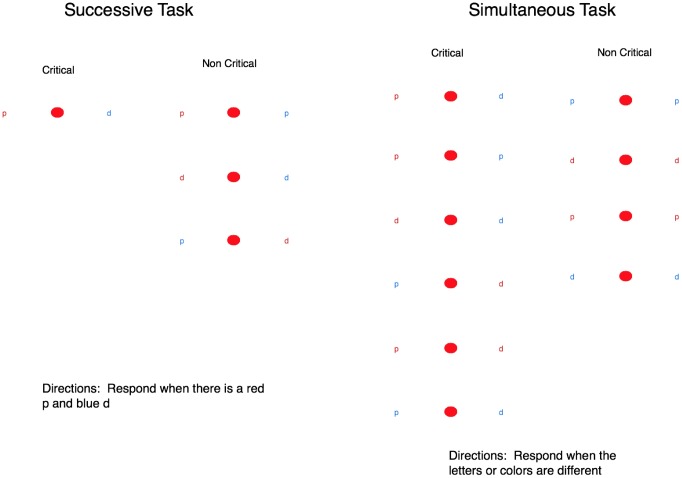
The left image shows the critical and neutral stimuli for the successive task and the right image shows the critical and neutral stimuli for the simultaneous task. These images are not to scale and the letters appeared in different locations each trial. The color of the letter and the type of letter were randomized across the locations.

Simultaneous and successive tasks were defined to require integrating information about both the color and letter identity of the stimulus elements (see **Figure 1**). For the simultaneous task, a critical trial was defined as instances where either the letters or the colors differed between the stimulus elements. For the successive task, critical stimuli comprised a red “p” and a blue “d,” requiring the participant to discriminate both the color and the type of letter to identify critical trials. For the successive task, the letters always differed in color within a trial to ensure that participants could not use peripheral vision to determine some non-critical trials by simply detecting whether or not the stimuli were the same color. Critical trials in the successive task included different colored stimuli, but this was not the case for the simultaneous task because we emphasized the requirement of making a color and letter judgment in both task conditions.

The successive and simultaneous vigilance tasks were similar to the task used by Hitchcock et al. (1999) in that participants were asked to take on the role of an air traffic controller. The instructions told participants that different combinations of letters represented impending vehicle collisions that required the participant to press the <SPACEBAR> key to be prevented; but if the letters did not indicate a collision, then no response was required. Each task had 1200 trials that lasted for 2 s each. The 1200 trials were divided into four blocks, although the division of trials into blocks was not signaled to participants. Each block consisted of 300 trials that had 10 critical trials for a total of 40 critical trials during the 40-min period. Critical trials were randomly distributed, with the constraints that there be 10 critical trials within each 10-min block and that critical trials could not occur consecutively.

**FIGURE 2:**
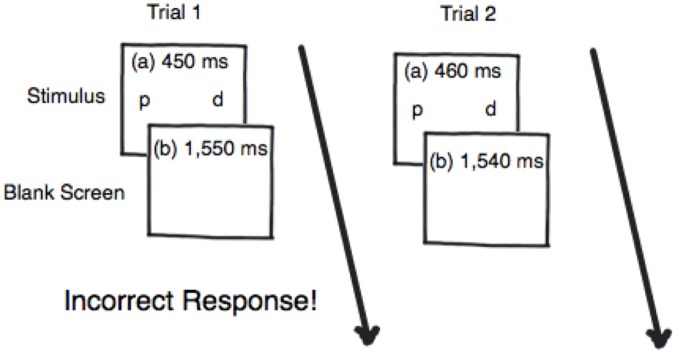
An example of the thresholding procedure where when the participant responds incorrectly to Trial 1 the signal duration for Trial 2 is presented for 10 ms longer.

Before beginning the experiment, participants were pretested to tailor stimulus presentation times to individual differences in performance. This was accomplished using a 10-min 3-down:1-up staircase thresholding procedure, which was also used as the training session (Leek, 2001). The thresholding procedure began with the stimuli appearing for 450 ms (see **Figure 2**). If the participant identified three critical trials correctly in a row then the stimuli presentation time decreased by 10 ms. However, if the participant made an error or missed a critical trial then the stimuli presentation time increased by 10 ms. Critical trials appeared 25% of the time during the thresholding procedure. There were 300 trials, resulting in 75 critical trials for thresholding. The thresholding procedure was incorporated to ensure that all participants began the task at the same performance level and to ensure that performance differences were due to the simultaneous/successive task distinction – as opposed to task or individual differences. No participants were eliminated based on poor performance on the thresholding procedure since the procedure was designed to equate baseline performance.

#### Design and Procedure

Participants were randomly assigned to either the successive or simultaneous task condition and tested individually. When the participants arrived at the lab, they were shown to a small experiment room with florescent lighting and told that they would be taking on the role of an air-traffic controller. They were seated approximately 66 cm from the computer monitor. Participants were not told how long the experiment would last. They were then calibrated on an SMI eye tracker operating at 250 Hz. Instructions were given to participants on how to complete the task, followed by 10-min of the thresholding procedure where participants received auditory feedback on correct or incorrect responses.

The stimulus duration for the main task was then determined based on the outcome of the thresholding task. The resulting mean stimulus duration for the successive task was 556.11 ms. For the simultaneous task condition, it was 522.03 ms. Before beginning the main portion of the experiment, participants were shown the instructions on the task again. This was followed by 1200 trials of the vigilance task where each trial of the task lasted for 2 s, for a total time-on-task of 40 min. During the main task, no feedback was provided to participants on correct or incorrect responses, the participants cell phone was taken away, and the experimenter left the room. The experiment was presented to participants using EPrime 2.0 (E-Prime 2. 0 [Computer software], 2012).

#### Measures

Keystroke data were collected for each participant to evaluate the accuracy and latencies of responses. In addition, eye tracking data was collected to determine the location and timing of fixations during each trial. Fixations were determined using the dispersion-based method, where fixations were defined by a sequence of point-of-regard samples spanning at least 30 ms that all fell within a 50-pixel radius. This represents relatively liberal criteria for defining a fixation. We opted for this because the stimuli in this task required the participant to make very quick eye movements to accurately encode the stimuli to make a correct judgment on each trial.

### Results and Discussion

#### Results of Performance Data

Before the experiment began, a thresholding procedure was used to equate the processing time of the successive and simultaneous tasks. A *t*-test determined if there were differences in the required signal duration for each participant to achieve an equivalent level of performance in the two tasks. The trend was for the successive task to require longer stimulus durations than the simultaneous task (*M* = 556 ms, *SD* = 78.60 for the successive task; *M* = 522 ms, *SD* = 82.85 for the simultaneous task), but this difference was not significant, *t*(55) = 1.59, *p* = 0.12, *d* = 0.42.

A 2 × 4 (task by block) mixed analysis of variance (ANOVA) was run for both critical and neutral trials. Based on Gartenberg et al. (2015), block was analyzed as a numeric factor rather than a categorical factor, as is common in the vigilance literature. This decision impacts the degrees of freedom relative to the more typical approach in the vigilance literature, which treats block as a categorical factor. Our analysis prevents an improvement from block to block from contributing to the vigilance decrement effect. We conducted the analysis both with and without a response cutoff for critical trials.

**FIGURE 3:**
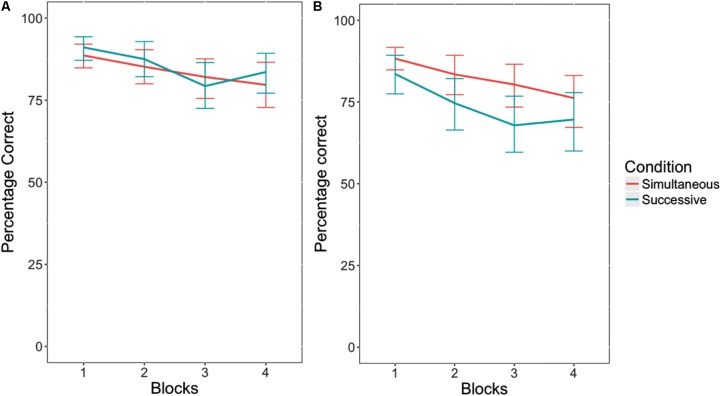
Accuracy in responding to critical trials in the simultaneous versus successive versions of the vigilance task, without a response cutoff **(A)**, and with a response cutoff **(B)**. Error bars indicate 95% confidence intervals.

When no response cutoff was used, there was a significant vigilance decrement, *F*(1,55) = 13.46, *p* < 0.05, η^2^ = 0.24, where participants performed worse as the blocks progressed (see **Figure 3A**). There was no effect of task type on critical trial performance, *F*(1,55) = 0.25, *p* = 0.62, η^2^ = 0.00. This indicates that the successive task did not exhibit a steeper decline in accuracy across blocks than the simultaneous task. In addition, there was no interaction between task type and block, *F*(1,55) = 0.00, *p* = 0.97, η^2^ = 0.00.

Interestingly, when a 1500 ms response cutoff was applied, as is typical when analyzing vigilance tasks similar to the one used in this experiment (Hitchcock et al., 1999, 2003), there was a significant difference in accuracy between task types, *F*(1,55) = 4.31, *p* < 0.05, η^2^ = 0.08. Participants performed worse in the successive task condition than the simultaneous task condition (see **Figure 3B**). Similar to when there was no response cutoff, there was an effect of block. Participants performed worse on both tasks as the blocks progressed, *F*(1,55) = 20.90, *p* < 0.05, η^2^ = 0.38. However, there was no interaction between the memory load conditions and block, *F*(1,55) = 0.23, *p* = 0.63, η^2^ = 0.00.

For neutral trials, there was no effect of task type, *F*(1,55) = 0.38, *p* = 0.55, η^2^ = 0.01. Surprisingly, performance improved as block increased, *F*(1,55) = 18.79, *p* < 0.05, η^2^ = 0.34. This reflects an overall trend toward fewer responses with time on task, since fewer correct detections were made in later blocks as well. There was no interaction between the memory load conditions and block, *F*(1,55) = 0.06, *p* = 0.81, η^2^ = 0.00 (see **Table 1**). To explore this trend more deeply, we computed A′ and β for each condition and each block (**Table 2**) to evaluate changes in bias and sensitivity, respectively (c.f., Grier, 1971; Stanislaw and Todorov, 1999; Thomson et al., 2016). The analysis indicates that both factors may have been influencing the performance changes observed in the task. There was both a significant change across blocks in β, *F*(1,55) = 17.84, *P* < 0.001 as well as a change in sensitivity, as changes in A′ were significant as well, *F*(1,55) = 11.29, *P* < 0.01, even though changes in A′ were modest. There was no effect of memory load condition in either case (*p* > 0.4), and no interaction (*p* > 0.9).

**Table 1:** Mean percent correct for neutral trials as a function of task condition and block number.

	Simultaneous task Mean (*SD*)	Successive task Mean (*SD*)
Block 1	97.99% (1.75%)	98.19% (1.21%)
Block 2	98.18% (1.64%)	98.41% (1.33%)
Block 3	98.36% (1.58%)	98.62% (1.21%)
Block 4	98.66% (1.17%)	98.77% (1.08%)

**Table 2:** A-prime and beta measures for each task condition and block.

	Simultaneous task		Successive task	
	A′	β	A′	β
Block 1	0.965	3.963	0.972	3.629
Block 2	0.956	5.179	0.963	5.174
Block 3	0.948	6.400	0.943	8.114
Block 4	0.944	8.213	0.954	7.749

**FIGURE 4:**
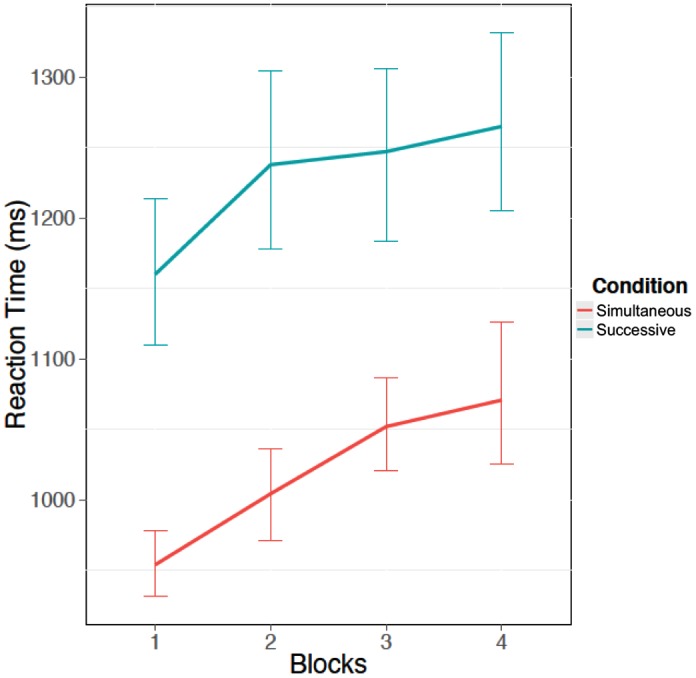
Mean reaction time (ms) for critical trials over the four blocks of the vigil for both successive and simultaneous tasks. Error bars indicate 95% confidence intervals.

To further explore the role of the response cutoff in the task type effect, we examined the reaction time to critical signals. Recall that it was hypothesized that participants would take longer to respond in the successive task condition than the simultaneous task condition due to the impact of the memory imperative in the successive task on the information processing requirements. Again, we ran a 2 × 4 mixed ANOVA with block as a numeric factor. There was a significant increase in reaction time as block increased, *F*(1,55) = 41.36, *p* < 0.05, η^2^ = 0.75 (**Figure 4**). In addition there was a main effect of task type, where participants responded more slowly in the successive task than the simultaneous task, *F*(1,55) = 45.60, *p* < 0.05, η^2^ = 0.83 (see **Figure 4**). However, there was no interaction between the memory load conditions and block, *F*(1,55) = 0.44, *p* = 0.51, η^2^ = 0.01.

#### Discussion of Performance Data

We used a novel task paradigm to ensure that participants in the successive and simultaneous task conditions had to look at two stimuli to make a critical judgment, and we implemented a thresholding procedure to ensure that the stimulus presentation times were equated across task type conditions based on the information processing requirements. Additionally, we analyzed the data with and without a response cutoff. These methodological choices were made to provide new evidence to understand differences in performance that have been reported for simultaneous and successive tasks in the vigilance literature.

From the perspective of Resource Theory, it has been argued that successive task performance is typically worse than in simultaneous tasks because the additional memory imperative results in depleting multiple resources (Davies and Parasuraman, 1982; See et al., 1995). We found that when no cutoff was used, there was no difference in accuracy between successive and simultaneous tasks, including changes with time on task. However, when a response cutoff was used, there was a difference in critical trial accuracy between tasks, where participants performed worse in the successive task. This raises questions about how to properly characterize the role of memory in task type performance differences.

The findings are consistent with Resource-Control Theory and our computational model. Disruptions in goal-directed cognitive processing will produce slowdowns that increase the likelihood that response time will exceed a threshold. They do not, however, produce differences in perceptual sensitivity to critical stimuli as a function of memory engagement. The findings suggest that a single attentional resource may be sufficient to explain the vigilance decrement, including the successive/simultaneous task distinction. The reaction time data reinforce these accounts because they show slowed performance, in addition to reduced accuracy, as time-on-task increased. Moreover, the slowing was similar for both the successive and simultaneous tasks, suggesting a similar degree of decline for both conditions.

In addition to the theoretical contributions, our findings have methodological implications for the practice of using a response cutoff for critical trials (Hitchcock et al., 2003; Szalma et al., 2006). This study shows that using a response cutoff can result in overestimation of the consequences of the vigilance decrement on performance accuracy. This may lead to results that are not attributable to differential changes in cognitive performance, but rather are a function of how degraded cognition interacts with the processing requirements for the task. The methodological decision to institute a response cutoff is one possible explanation as to why other researchers have reported that the vigilance decrement is more severe for successive tasks than simultaneous tasks – and it may impact other vigilance findings in the literature as well.

#### Eye Tracking Results

Eye tracking data provide a continuous stream of data that is informative with respect to the focus and content of current cognitive processing. In the context of this study, eye data expose when items are attended and encoded, helping to divide individual trials into stages. We use the eye tracking data to identify when each stimulus element was encoded, and when decisions were made about whether or not to respond. Importantly, the model in Veksler and Gunzelmann (2018) makes explicit predictions about the cognitive processes involved while performing a sustained attention task. The model posits that microlapses may occur at any stage of processing the stimulus, and that the likelihood of microlapses increases within individual trials, in addition to increases emerging over the course of the experiment session. Because of the within-trial fluctuations, there should be a greater impact of the vigilance decrement in later stages of processing within a trial (e.g., between encoding and responding). In addition, the involvement of memory in the successive task means that it should take longer to process the stimuli once one or both stimulus elements are encoded because this is where the memory impacts the information processing demands for performing the task.

To explore these hypotheses, eye movement data was used to examine how participants processed the stimulus within a trial, and how that processing changed over the course of the vigilance task. The eye movement data was used to divide each trial into three segments: (a) The time period between stimulus presentation and the first fixation on a stimulus element, (b) the time between the first fixation on the first stimulus element and the first fixation on the second stimulus element, and (c) the time between looking at the second stimulus element and responding (on trials where responses were made). For this analysis, no response cutoff was used. A fixation was counted as being on the second stimulus if the first fixation after the stimulus disappeared was on the second stimulus location.^1^

Our prediction is that participants will take longer on all stages of processing as time on task increases. One consequence of this should be that participants become less likely to look at both stimulus elements in a trial over the course of the experiment, leading to errors. We posit that this kind of error, which we refer to as Processing Time Errors, or PTEs, is a major source of errors in sustained attention tasks (Gartenberg, 2016). In addition, increases in the duration of later stages of processing within trials (e.g., between encoding and responding) should be larger than changes observed in earlier stages (e.g., between stimulus presentation and fixating on the first stimulus element).

A mixed ANOVA was run on the duration of the three trial segments, where block was again a numeric within-subjects variable and condition was a between-subjects variable. Three participants were eliminated from the analysis on the third stage of processing because they had no responses for a full block. Trials where participants did not look at the second stimulus were not included in the analysis of second segment times. Likewise, trails were only included for the third time segment if participants responded.

**FIGURE 5:**
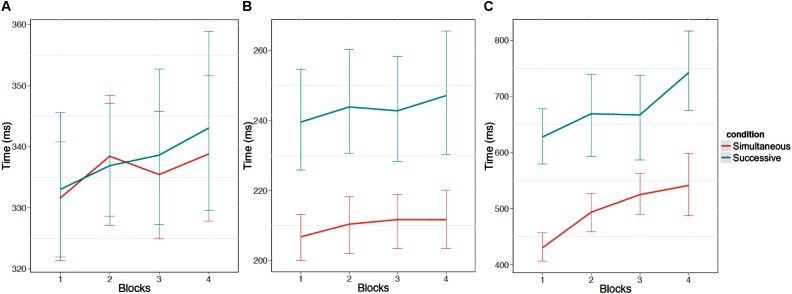
Times (ms) for each stage of stimulus processing. **(A)** Shows the time to fixate the first stimulus element. **(B)** Show the time between the first fixation of the first stimulus element and the first fixation on the second stimulus element. Finally, **(C)** shows the time between the first fixation on the second element and the response. Error bars are 95% confidence intervals.

**FIGURE 6:**
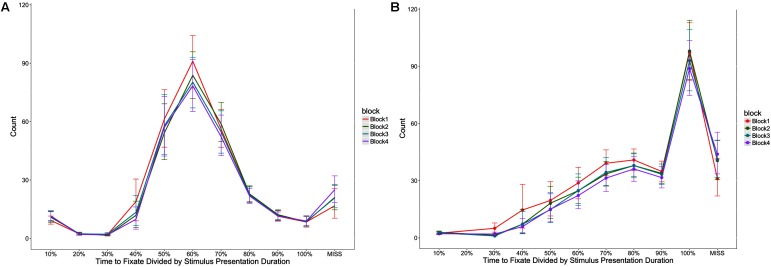
Distribution of trial time taken to fixate the first **(A)** and second **(B)** stimulus elements, represented as a percentage of the total stimulus presentation time. In **(B)**, the percentage reflects the cumulative time for fixating both stimulus elements. The *y*-axis represents the number of trials recorded where each proportion of time was take to fixate the stimulus elements. Error bars indicate 95% confidence intervals.

Participants took longer to look at the first stimulus element as block increased, *F*(1,52) = 8.50, *p* < 0.05, η^2^ = 0.16, took longer between fixating the first and second stimulus elements as block increased *F*(1,52) = 7.63, *p* < 0.05, η^2^ = 0.15, and took longer to respond after fixating the second stimulus element as block increased, *F*(1,49) = 25.37, *p* < 0.05, η^2^ = 0.52 (see **Figure 5**). **Figure 6** provides an additional illustration of this effect. It demonstrates how the distributions of the time taken to fixate successive stimulus elements and then respond increased across the four blocks. The consequence is that participants were less likely to fixate both stimulus elements in later blocks.

There was no effect of condition for the first stage of processing (i.e., how long it took to look at the first stimulus), *F*(1,52) = 0.05, *p* = 0.82, η^2^ = 0.00, but there was an effect of condition on looking at the second stimulus, where participants took a longer amount of time between looking at the first stimulus and the second stimulus for the successive task condition, *F*(1,52) = 16.33, *p* < 0.05, η^2^ = 0.31. This finding was further supported by the interval of time between looking at the second stimulus and responding, where participants took longer to respond once the second stimulus had been fixated in the successive task condition, *F*(1,49) = 41.42, *p* < 0.05, η^2^ = 0.85. This result reinforces the perspective that memory processes impact decision time, but not accuracy *per se*, in this task since memory involvement depends on information available only after the first stimulus is attended.

There was no interaction between the task type and block for any of the three intervals of time: looking at the first stimulus, *F*(1,52) = 0.60, *p* = 0.44, η^2^ = 0.01, looking at the second stimulus, *F*(1,52) = 0.17, *p* = 0.68, η^2^ = 0.00, and responding *F*(1,49) = 0.04, *p* = 0.85, η^2^ = 0.00. Once again, this reinforces the similarity of the decrements observed in both task types.

Participants looked at both stimulus elements less frequently as block increased, *F*(1,52) = 17.48, *p* < 0.05, η^2^ = 0.34. Not looking at both was defined by when participants either looked at only one stimulus or did not look at either stimulus. There was no effect of task type on the percentage of time that participants looked at both stimuli, *F*(1,52) = 1.71, *p* = 0.20, η^2^ = 0.03, suggesting that the thresholding procedure was effective. In addition, there was no interaction between task type and block, *F*(1,52) = 3.01, *p* = 0.09, η^2^ = 0.06, once again providing evidence that the declines in performance were similar for both tasks.

Importantly, for the successive task, participants did not always have to look at both stimuli for some neutral trials, since in some trials the first stimulus element could identify the stimulus as non-critical. As a result, participants may be less likely to look at the second stimulus in the successive task condition. However, we found that participants typically looked at both stimuli in both task type conditions and that there was not a significant difference in the percentage of time that participants looked at the second stimulus for the successive task (*M* = 81.94%, *SD* = 17.61%) as compared to the simultaneous task (*M* = 86.65%, *SD* = 10.02%), *t*(52) = -1.22, *p* = 0.23.

#### Results of Eye Data Stage of Processing Analyses

To further explore how the stage of processing the stimuli impacted the rate of slowing over time, the stage of processing was added to the mixed ANOVA model. Stage of processing was included in the model as a within group variable and each stage of processing was compared to one another, including: looking at the first stimulus element, the time between looking at the first stimulus element and the second stimulus element, and the time between looking at the second stimulus element and responding.

When comparing the first stage of processing with the second stage of processing, consistent with the previous results, there was an effect of task type where participants were slower in the successive task condition than the simultaneous task condition, *F*(1,49) = 9.67, *p* < 0.05, η^2^ = 0.20, there was an effect of block where more slowing occurred as block progressed, *F*(1,49) = 6.37, *p* < 0.05, η^2^ = 0.13, and there was no interaction between task type and block, *F*(1,49) = 0.04, *p* = 0.85, η^2^ = 0.00. There was a main effect of stage of processing where overall, participants were slower for the first stage of processing than the second stage of processing, *F*(1,49) = 224.56, *p* < 0.05, η^2^ = 4.58. There was an interaction between task type and stage of processing where participants were slower in the second stage of processing for the successive task condition, *F*(1,49) = 13.72, *p* < 0.05, η^2^ = 0.28. There was no interaction between block and stage of processing, *F*(1,49) = 0.06, *p* = 0.80, η^2^ = 0.00, and no three-way interaction between task type, block, and stage of processing was observed, *F*(1,49) = 0.01, *p* = 0.91, η^2^ = 0.00. This indicates that the degree of within-trial slowing was similar for both tasks.

The second stage of processing was then compared with the third stage of processing. Again, there was an effect of task type where participants were slower in the successive task condition than the simultaneous task condition, *F*(1,49) = 50.90, *p* < 0.05, η^2^ = 1.04, there was an effect of block where more slowing occurred as block progressed, *F*(1,49) = 29.14, *p* < 0.05, η^2^ = 0.59, and there was no interaction between task type and block, *F*(1,49) = 0.06, *p* = 0.81, η^2^ = 0.00. There was a main effect of stage of processing where overall, participants were slower for the third stage of processing than the second stage of processing, *F*(1, 49) = 519.85, *p* < 0.05, η^2^ = 10.61. There was an interaction between task type and stage of processing where participants were slower in the third stage of processing for the successive task condition, *F*(1,49) = 20.67, *p* < 0.05, η^2^ = 0.42. Unlike the comparison between the first stage of processing and the second stage of processing, there was an interaction between block and stage of processing where more slowing across blocks occurred in the third stage of processing than the second stage of processing, *F*(1,49) = 15.82, *p* < 0.05, η^2^ = 0.32. This provides some support for the model prediction that degradations are compounded within a trial to produce larger deficits in later stages with time on task. There was no three-way interaction between task type, block, and stage of processing, *F*(1,49) = 0.01, *p* = 0.92, η^2^ = 0.00.

A similar pattern of results was found when comparing the first stage of processing with the third stage of processing, where there was increased slowing over time for the third stage. Again, there was an effect of task type where participants were slower in the successive task condition than the simultaneous task condition, *F*(1,49) = 42.21, *p* < 0.05, η^2^ = 0.86, there was an effect of block where more slowing occurred as block progressed, *F*(1,49) = 27.39, *p* < 0.05, η^2^ = 0.56, and there was no interaction between task type and block, *F*(1,49) = 0.04, *p* = 0.84, η^2^ = 0.00. There was a main effect of stage of processing where overall, participants were slower for the third stage of processing than the first stage of processing, *F*(1,49) = 254.1, *p* < 0.05, η^2^ = 5.19. There was an interaction between task type and stage of processing where participants were slower in the third stage of processing for the successive task condition, *F*(1,49) = 25.60, *p* < 0.05, η^2^ = 0.73. Similar to the comparison between the second stage of processing and the third stage of processing, there was an interaction between block and stage of processing where more slowing across blocks occurred in the third stage of processing than the first stage of processing, *F*(1,49) = 19.20, *p* < 0.05, η^2^ = 0.39. Again, this supports the prediction that slowing over time should be greater in later stages of processing within a trial. No three-way interaction between task type, block, and stage of processing was observed, *F*(1,49) = 0.01, *p* = 0.92, η^2^ = 0.00.

#### Discussion of Eye Data

The eye data provide further insight into the degradations responsible for the vigilance decrement. At each stage of processing, participants took longer as block (i.e., time-on-task) increased over the course of the sustained attention tasks. This result is consistent with the hypothesis that degradations in goal-directed cognitive processing accumulate over the course of time on task, and also that these degradations occur throughout the processing of a trial. As a result, as time-on-task increases, participants are less likely to look at both stimuli, leading to *processing time errors*. These findings also support our model, which asserts that microlapses cause cognitive slowing, which can interfere throughout the process of encoding trial information and responding (Gartenberg et al., 2014; Veksler and Gunzelmann, 2018; see also Gunzelmann et al., 2012).

The eye data supported the hypothesis that it takes longer to process the stimulus in successive tasks than simultaneous tasks due to the additional memory imperative of successive tasks. Specifically, the eye data show that this effect is focused on portions of the trial following the encoding of the first stimulus element. There was no difference between successive/ simultaneous tasks for how long it took participants to look at the first stimulus element, suggesting that the processing is initially similar in the two tasks. However, at later stages of the trial, including looking at the second stimulus and responding, participants took a longer amount of time for the successive task.

Finally, the results provide some support for the hypothesis that within-trial declines lead to larger increases in processing time for later stages of the trial. Larger increases were observed for the third stage of trials than for the first or second. This is consistent with Veksler and Gunzelmann (2018), which proposes that degradations in performance in vigilance tasks should be more pronounced in later stages of stimulus processing and responding. Importantly, the magnitude of the changes was similar for both task types, providing evidence for a common mechanism being responsible for the vigilance decrement in both cases. This result is consistent with both Resource Control Theory and with Veksler and Gunzelmann (2018).

## General Discussion

We explored the role of memory in sustained attention tasks to test the hypothesis that depletion of central executive attentional resources is sufficient to explain results for both the successive and simultaneous tasks documented in the literature (Davies and Parasuraman, 1982; See et al., 1995). We designed simultaneous and successive tasks where we controlled for visual encoding requirements and adjusted stimulus presentation times to equate the task conditions by using a thresholding procedure. In addition, we collected eye tracking data to gather evidence about how cognitive processing unfolds over the course of performing each trial. We also assessed the impact of response cutoffs on the magnitude of the vigilance decrement and differences between simultaneous and successive variants. Finally, we treated block as a numeric variable in our ANOVA model to appropriately treat that variable in our analyses. With these methodological adaptations, we found no difference in the magnitude of the vigilance decrement between simultaneous and successive tasks in our study.

The eye movement data provide further evidence regarding differences between simultaneous and successive tasks. There was no difference in how long it took participants to look at the first stimulus, yet participants performing the successive task took longer in later stages of processing. This is consistent with the expectation that the need for declarative knowledge will influence task performance after the stimulus is encoded and can be a major influence on results that have been documented in the vigilance literature. However, consistent with Veksler and Gunzelmann (2018), differences in the magnitude of the vigilance decrement between task types may not be the caused by depleting an additional memory resource as is often theorized, but instead may arise from degradations in attentional control that produce more significant impacts on performance in some cases depending on the task design and information processing requirements.

Our results provide useful evidence regarding current theories of the vigilance decrement, although they do not decisively settle the debates in this domain. The lack of a difference in the vigilance decrement between simultaneous and successive tasks indicates that a single resource may be sufficient. This possibility challenges at least some versions of Resource Theory. Meanwhile, the results do not support the conclusion that performance changes stem from mindlessness or boredom. If boredom were the issue, it would seem that breakdowns ought to be larger for earlier stages of individual trials, in contrast to the observed effect where deficits accumulated within trials. The finding that the largest degradations came in the final stage of processing suggests that participants were sufficiently engaged to attend to relevant stimulus elements when they appeared. Much of the deficit appears to result from a need for increased time to process the stimulus at each stage, especially the final stage where a response is generated. This pattern of results argues against some common claims advanced by proponents of these theories.

The results are consistent with Resource-Control Theory and with Veksler and Gunzelmann (2018). In both cases, the changes in performance are interpreted as arising from depleted cognitive resources, and mindlessness is implicated as the primary consequence of diminished cognitive control. In Veksler and Gunzelmann (2018), time on task impacts processes that control the selection and execution of cognitive actions. As the degradations accumulate, the primary result is a reduction in the signal-to-noise ratio in this process, leading to an increased probability that goal-directed processing will fail. In the model, breakdowns of 10’s of ms occur more frequently with time on task, leading to delays in task execution. As observed in this study, these delays ultimately produce *processing time errors*, where stimuli are not fully encoded during the presentation time, preventing accurate performance on the task. More research is needed to investigate the predictions of this model and Resource-Control Theory, to develop a more detailed and comprehensive account of the vigilance decrement.

The primary empirical result of this study was the observation that there was no difference in critical trial performance between tasks when no response cutoff was used, but worse performance for the successive task condition when a cutoff was instituted. The convention when analyzing many vigilance tasks is to use a response cutoff (Hitchcock et al., 1999; Hitchcock et al., 2003; Szalma et al., 2006). This is typically done to avoid misattributing false alarms as correct responses. However, our results suggest that the greater risk may be overestimating the negative consequences of the vigilance decrement on accuracy, particularly in tasks that require more complex processing.

Our findings have important implications for vigilance research beyond the simultaneous/successive task distinction. The standard practice of instituting a response cutoff could impact a number of effects in the sustained attention literature, such as signal saliency effects and modality effects. The implications for applied domains may be even more critical, however. The results indicate that relatively minor manipulations of task requirements and data processing can have substantial influences on the consequences of reduced vigilance with time on task. Creating real-world systems that are more robust to the vigilance decrement in their human operators is a topic worthy of additional research to reduce the likelihood of critical errors.

## Ethics Statement

This study was carried out in accordance with the recommendations of the Human Subjects Research Guidelines from the George Mason University Institutional Review Board (IRB), with written informed consent from all subjects. All subjects gave written informed consent in accordance with the Declaration of Helsinki. The protocol was approved by the George Mason University IRB.

## Author Contributions

GG consulted on the design of the study and interpretation, and led authorship of the manuscript in preparation for this submission. DG led the study design, analysis of the data, and initial write-up of methods and results. SH-B led data collection. JT contributed to all aspects of the research. All authors contributed to the manuscript.

## Conflict of Interest Statement

The authors declare that the research was conducted in the absence of any commercial or financial relationships that could be construed as a potential conflict of interest.

## Footnotes

1 The second stimulus fixation was calculated this way because there was no mask and an exploratory data analysis demonstrated that participants frequently looked at the second stimulus after the stimulus disappeared yet were often correct on the trial (see **Appendix A**). This may be due to after image effects that can occur without a mask.

## APPENDIX A

In the figure below, the gray/black gradient dots are samples from the 250 Hz eye tracker. The green ‘B’ indicates when the samples began and the red ‘E’ represents when the samples ended. The red dots represent fixations that occurred during the stimulus presentation and the orange dots represent fixations that occurred after the stimulus presentation. As can be seen in this example, the participant got this trial correct, but looked at the second stimulus after it disappeared, where the stimulus presentation time (e.g., dwell time) was 600 ms, but they looked at the second stimulus at 664.48 ms. Since participants should not be able to answer correctly without looking at both stimuli, the first fixation after the stimulus disappeared was included as a fixation on the second stimulus.
